# Norcantharidin Induces HL-60 Cells Apoptosis In Vitro

**DOI:** 10.1155/2012/154271

**Published:** 2012-06-24

**Authors:** You-Ming Jiang, Zhen-Zhi Meng, Guang-Xin Yue, Jia-Xu Chen

**Affiliations:** ^1^School of Pre-Clinical Medicine, Beijing University of Chinese Medicine, Beijing 100029, China; ^2^Institute of Basic Theory of TCM, China Academy of Chinese Medical Sciences, P.O. Box 83, Beijing 100700, China; ^3^Department of Basic Theory in Chinese Medicine, Henan University of Traditional Chinese Medicine, Zhengzhou 450008, China

## Abstract

Norcantharidin (NCTD) is the demethylated form of cantharidin, which is the active substance of mylabris, and is known to have anticancer potentials. The aim of this paper was to assess the apoptosis-inducing effect of NCTD on HL-60 cells. *Methods*. The effects of NCTD were detected by flow cytometer on the cell toxicity, cell cycle, and apoptosis of HL-60 cells cultured in vitro. *Results*. After 48-hour treatment with NCTD, the growth of HL-60 cells was inhibited significantly. The summit of apoptosis appeared after 24 hours. The percentage of the cells in G_1_ phase decreased and then increased in S and G_2_ *+* M phase, while the S and G_2_ *+* M phases were blocked after treatment with 5, 10, and 50 **μ**mol/L NCTD for 24 hours. *Conclusions*. NCTD can induce the apoptosis of HL-60 cells and inhibit the fissiparism, and the domino effect was obviously correlated with the time and dosage.

## 1. Introduction

 Mylabris, the polypide of Mylabris phalerata Pall or Mylabris cichorii Linnaeus, characterized by being cold in nature, acrid in flavor, and toxic, has the effect of removing toxic substance for cellulites, breaking blood stasis, and dispersing accumulation. It has been showed that cantharidin is the main ingredient for anticarcinogenic effect of mylabris, chemical structure of which is exo-1,2-syn-dimethyl-3,6-oxidohydrophthalic anhydride. Norcantharidin (NCTD) is the demethylated form of cantharidin, which is the active substance of mylabris, and is known to have anticancer potentials. NCTD is a kind of new-type anticancer drug with the effect of increasing white cells. It was synthesized with furan and maleic anhydride through Diels-Alder reaction [1] and was currently used as an anticancer drug in China. Many experiments including our previous study [2] have demonstrated that NCTD can inhibit the growth of tumor cells in vitro and in vivo [3–9]. However, the exact anticancer mechanism of NCTD on human cancer cells remains poorly understood. In the present study, flow cytometer and cytomorphology staining were used to study the effect of NCTD on apoptosis and cell cycle of HL-60 cells. 

## 2. Materials and Methods

### 2.1. Cell Strain

HL-60 cells were cultivated in pure RPMI-1640 (purchased from GBICO), placed in a temperature-controlled CO_2_ incubator (37°C), transfer of culture one time every 2-3 days. Experiment started when cells entered exponential growth stage, the best state.

### 2.2. Drugs and Agent

Calf serum was provided by Institute of Hematologic Disease in Tianjin affiliated to Chinese Academy of Medical Sciences; NCTD was purchased from the Forth Pharmaceutical Factory in Beijing and was dissolved by DSMO. RNA enzyme was purchased from Huamei Company, the concentration of which used in this experiment was 0.02 g/L. Propidium iodide (PI) (Sigma Company), the concentration of which used in this experiment was 0.05 g/L. 

### 2.3. Flow Cytometer

Fluorescence-activated cell sorter, being manufactured by USA Becton Dickinson Company, type of 420, was provided by Institute of Basic Theory, China Academy of TCM.

### 2.4. Experiment of Dose and Time Response on HL-60 Cells by NCTD

Cell concentration was adjusted into 5×10^8^/L when HL-60 cells were at the logarithmic increasing phase; then the cells were inoculated on 12-well plates. Each well added 1 mL cell suspension and 3 mL RPMI-1640 containing 10% calf serum, then added NCTD (the final concentrations were 5 *μ*mol/L, 10 *μ*mol/L, 50 *μ*mol/L, resp.), and DSMO as control group; each group had 3 parallel wells. Specimens were collected at 24 h, 48 h after the cell suspension was cultured in temperature-controlled incubator (37°C, 0.05 CO_2_). Adding 200 *μ*L cell suspension into 100 *μ*L 1.8% NaCl and 100 *μ*L 4% trypan blue stock solution to stain. Stained specimens were put into centrifugal machine for 1 min at 1000 r/min, sucking the supernatant. The supernatant was washed by PBS (pH 7.4, 0.05 mol/L) two times and centrifuged for 1 min at 1000 r/min to count the alive cells and dead cells under the invert microscope after the colorless supernatant was dropped on slide.

### 2.5. Experiment of Dose and Time Response on HL-60 Cells Apoptosis and Cell Cycle by NCTD

The concentration and action time of NCTD were the same as mentioned above. To collect cells after medication treatment, wash the cells with PBS not containing Ca^2+^, Mg^2+^ and centrifuge them for 5 min at 800 r/min twice, respectively. Then use syringe with TB pinhead to infuse cells precipitation into 70% alcohol (4°C), shake until cells homogeneous dispersion, and fix the cells more than 18 h. Concentration of cells was adjusted into 1 × 10^9^/L after being centrifuged and washed. 500 *μ*L cell sap were lucifuge strained with 50 *μ*L 0.1% PI (50 mg/L Propidium iodide, 0.1% Sodium-cit-rate, 0.1% triton X-100) for 30 min. Specimen was filtered with 400 holes net, exciting wavelength was set at 488 nm, and blocking filter was 585 nm. Photomultiplier tube and multichannel pulse analyzer were used to show apoptosis scatterplot. Flow cytometer analysis showed that near diploid cell mass peak appeared at the left of G_1_ phase during cell apoptosis. Flow cytometer was used to determine the change of percentage of cell at G_1_ phase, S phase, and G_2_ + M phase. Each group had 1 × 10^6^ cells and 3 parallel wells.

### 2.6. Observation on Cytomorphology of HL-60 Cells after NCTD

The apoptotic morphology was observed by using staining reagent Wright-Giemsa. Collected HL-60 cells treated with NCTD at 1000 r/min for 5 min used PBS to rinse the collected cells one time. Collected cells were then resuspended in PBS and dropped on slide. Collected cells were fixed in Colonial spirit for 2-3 min after air drying, then open-air drying. Collected cells were stained in Giemsa stain. The stained cells were separated according to color in 95% alcohol for 30 s and dehydrated in absolute alcohol for 10–30 s, cleared in dimethyl benzene, and enveloped with neutral gum. Cellular shape was observed and photographed under type 2 Leitz-ORTHOLUX light microscope (German).

### 2.7. Statistical Method

Data were analyzed by analysis of variance (ANOVA) followed by post hoc *t* tests for comparisons between groups. Significance was accepted as *P* < 0.05. Data are expressed as mean ± standard deviation (SD).

## 3. Results

### 3.1. Dose and Time Response on HL-60 Cells by NCTD

**Figure 1:**
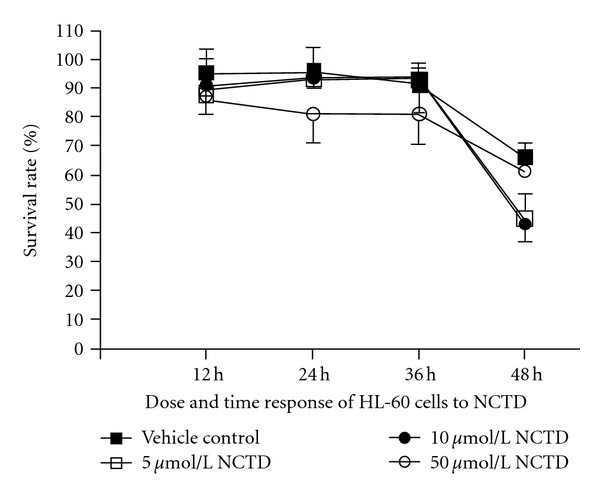
Cytotoxic effect of NCTD on HL-60 cells.

Trypan blue staining showed that the cytotoxic effect of NCTD on HL-60 cells increased with increase of time (*P* < 0.01); survival rate of HL-60 cells decreased with increase of the concentration of NCTD (*P* < 0.05). Results showed that cytotoxic effect of NCTD on HL-60 cells was time and dose concentration response (Figure 1).

### 3.2. Dose and Time Response on HL-60 Cells Apoptosis by NCTD. 

**Figure 2:**
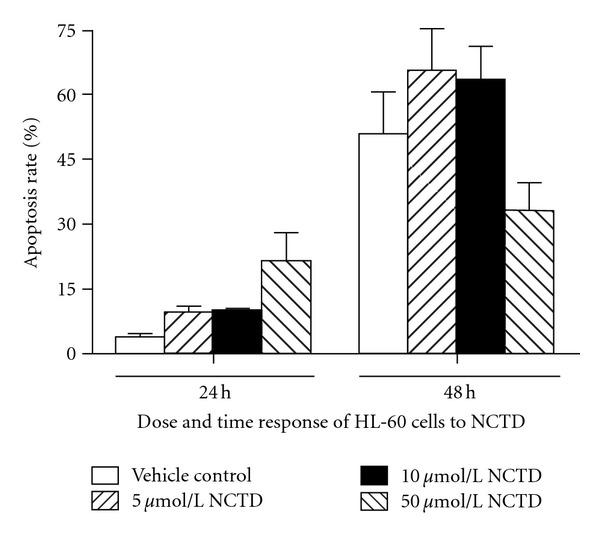
Effect of NCTD on HL-60 cells apoptosis.

Apoptosis of HL-60 appeared at 48 h (*P* < 0.01). Apoptosis of HL-60 induced by 5 *μ*mol/L NCTD for 48 h was more apparent than those induced by other concentrations and also was time dependent. Apoptosis of HL-60 induced by 50 *μ*mol/L NCTD for 24 h was obvious (Figure 2).

### 3.3. Effects of NCTD on Cell Cycle of HL-60 Cells. 

**Figure 3:**
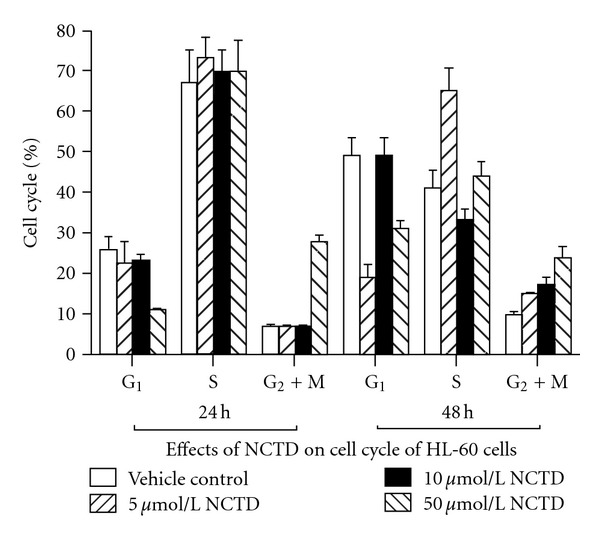
Effects of NCTD on HL-60 cell cycle.

Compared with blank control group, the percentages of HL-60 cells of G_1_ phase decrease after being treated by NCTD, while the percentages of HL-60 cells of S phase and G_1_ + M phase increased, the cell cycle was dramatically arrested at G_2_/M phase (*P* < 0.01). Effect of NCTD on HL-60 cells cycle was specially obvious with 50 *μ*mol/L for 24 h and 48 h (Figure 3).

### 3.4. Change of Cytomorphology of HL-60 Cells after NCTD Treatment

**Figure 4:**
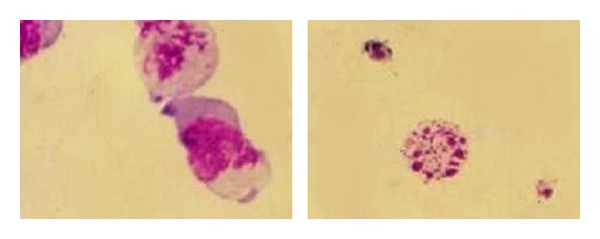
Cytomorphology of HL-60 cells after NCTD treatment (×500).

Under inverted microscope, the HL-60 cells growth was round. After treatment with NCTD, the cell growth was inhibited, the growth velocity decreased, and the cell changed from round into ellipse, horseshoe shape, gradually. There were several ecthyma on edge of cell membrane, the cell clarity decreased apparently. Above phenomena became more obvious with the increase of the concentration of NCTD. Wright-Giemsa staining showed that apparent change of morphology appeared after treating with different concentrations of NCTD for 24 h. Its expression was that the chromosome movement was abnormal at mitosis anaphase, nucleoplasm condensed into one or several big boluses, and cell nucleus split into shivers; thus ganoid apoptotic bodies encapsulated by cell membrane appeared to be intracellular (Figure 4 represents apoptosis cell and/or apoptotic body; left: 5 *μ*mol/L NCTD treatment group; right: 50 *μ*mol/L NCTD treatment group).

## 4. Discussion

 The development of tumor is closely related to apoptosis. An important foundation of tumor development is reinforcement of cell multiplication or apoptosis blocking or multiplication and apoptosis reinforcement, but the reinforcement of multiplication exceeded that of apoptosis significantly. In recent years, it has been paid attention to apoptosis induced by Chinese materia medica at home and abroad [10]. It has been proved that lots of anticancer drug originated from Chinese materia medica such as taxol [11], camptothecin, teniposide, harringtonine [12], and trichosanthin [13] can induce tumor cell apoptosis.

 Mylabris, characterized by being acrid in flavor, and cold in nature, is toxiferous; it should be processed before being used as decoction. Much attention has been attached to study on curative feasibility of toxiferous Chinese crude drug by the exploitation of its so-called function of fight fire with fire. Whether the mechanism of toxiferous Chinese crude drug is related to inducing apoptosis has been indefinite yet. 

 This study showed that the depressant effect of NCTD on HL-60 cells was apparent and had time and dose effect response. Flow cytometer showed that HL-60 cells appeared as apoptosis after treatment with 5 *μ*mol/L, 10 *μ*mol/L, 50 *μ*mol/L NCTD for 24 h and 48 h, and the effect of NCTD on HL-60 cells was dose dependent. After treating by NCTD, the percentage of HL-60 cells decreased at G_1_ phase, the percentage at S phase and G_2_/M phase increased, and the cell cycle was dramatically arrested at G_2_/M phase and S phase. It indicated that NCTD could inhibit the DNA synthesis of HL-60 cell at S phase obviously, interfere with karyokinesia, and thus, restrain the proliferation of tumor cells. In conclusion, NCTD can inhibit the growth of HL-60 cells by interfering with cell proliferation and inducing apoptosis. This has been proved by morphology staining, and its mechanism needs further investigation.
